# Performance of NUTRIC score to predict 28-day mortality in critically ill patients after replacing APACHE II with SAPS 3

**DOI:** 10.1371/journal.pone.0270455

**Published:** 2022-07-01

**Authors:** Ivens Augusto Oliveira Souza, Paulo Cesar Ribeiro, Joop Jonckheer, Elisabeth De Waele, Leandro Utino Taniguchi

**Affiliations:** 1 Intensive Care Unit, Hospital Sirio Libanes, São Paulo, São Paulo, Brazil; 2 Nutrition Support Team, Hospital Sirio Libanes, São Paulo, São Paulo, Brazil; 3 Intensive Care Department, UZ Brussel, Brussels Jette, Belgium; 4 Department of Nutrition, UZ Brussel, Jette, Belgium; 5 Vrije Universiteit Brussel, Brussels, Belgium; 6 Emergency Medicine Discipline, Hospital das Clinicas, São Paulo, São Paulo, Brazil; Azienda Ospedaliero Universitaria Careggi, ITALY

## Abstract

**Objectives:**

The *Nutrition Risk in the Critically Ill* (NUTRIC) score has been advocated as a screening tool for nutrition risk assessment in critically ill patients. It was developed and validated to predict 28-day mortality using *Acute Physiology and Chronic Health Evaluation II* (APACHE II) score as one of its components. However, nowadays the *Simplified Acute Physiology Score 3* (SAPS 3) demonstrates better performance. We aimed to test the performance of NUTRIC score in predicting 28-day mortality after replacement of APACHE II by SAPS 3, and the interaction between nutrition adequacy and mortality.

**Methods:**

Adult patients who received nutrition therapy and remained >3 days in intensive care unit were retrospectively evaluated. In order to replace APACHE II component, we used ranges of SAPS 3 with similar predicted mortality. Discrimination between these tools in predicting 28-day mortality was assessed using the ROC curve, calibration was evaluated with calibration belt, and correlation with intraclass correlation. The relationship between nutritional adequacy and mortality was assessed in a subgroup with available data.

**Results:**

542 patients were analyzed (median age of 78 years old, 73.4% admitted for non-surgical reasons and 28-day mortality was 18.1%). Mortality prediction discrimination did not differ between tools (p>0.05), but showed a good agreement (intraclass correlation 0.86) with good calibration. In the subgroup analysis for nutritional adequacy (n = 99), no association with mortality was observed.

**Conclusion:**

Performance of NUTRIC score with SAPS 3 is similar to the original tool. Therefore, it might be used in settings where APACHE II is not available.

## Introduction

Since malnutrition is prevalent in the acute hospital setting and associated with poor clinical outcomes [1], the latest American guidelines suggest nutritional risk assessment in all patients admitted to the intensive care unit (ICU) [2]. Such evaluation would allow for the appropriate adjustment of nutritional therapies, and identification of those most likely to benefit from early nutritional support. The *Nutrition Risk in the Critically Ill* (NUTRIC) score was originally proposed in 2011 as a tool to assess the relationship between nutritional risk and 28-day mortality in critically ill patients [3]. It has been validated by other authors [4], including a study applying a Portuguese version of the tool [5].

In the conceptual model of NUTRIC score, acuity would be one of mediators of worse clinical outcomes [3]. *Acute Physiology and Chronic Health Evaluation II* (APACHE II) score was originally included as such a severity-of-illness variable. However, nowadays APACHE II is outdated, and no longer accurate to represent acuity [6], and can only be calculated after 24 hours of ICU stay. Therefore, better performing severity-of-illness scores might be more adequate to update NUTRIC score. Moralez et al. recently demonstrated that *Simplified Acute Physiology Score 3* (SAPS 3), which is performed with information within the first hour of ICU stay, is the most accurate prognostic model in a multicenter Brazilian cohort [7]. Consequently, it is one of the best options.

Our study was designed to test the performance of NUTRIC after replacement of APACHE II by SAPS 3 (SAPS-NUTRIC) and the relationship between nutritional adequacy and 28-day mortality stratified by NUTRIC and SAPS-NUTRIC.

## Methods

### Study design and patient setting

This retrospective cohort study included adult patients (≥ 18 years old) admitted to the medical-surgical ICU of Hospital Sírio-Libanês (located in São Paulo, Brazil) between February 2016 and February 2019, who received enteral and / or parenteral nutrition therapy and remained > 3 days in the ICU. Exclusion criteria were pregnant women and patients with 48-hours death expectancy, palliative care or transferred from another hospital.

Cloud-based administrative (Epimed®) [8] and multidisciplinary nutrition therapy team databases were use as source of patient’s data, comprising demographic, clinical and body mass index (BMI); invasive organ supports (mechanical ventilation, use of vasoactive drugs, dialysis); organ failure (SOFA score) [9], nutritional therapy received during hospitalization (enteral, parenteral, amount of calories and protein); and clinical outcomes (discharges, deaths, transfers). The study was approved by the local institutional ethics committee (CAAE: 65382117.8.0000.5461), which waived informed consent due to the observational and retrospective design of the study.

### Scores calculation and study definitions

APACHE II score and SAPS 3 were calculated as originally described [10–12]. Of note, APACHE II uses the worst value of its parameters in the first 24 hours of ICU stay, while SAPS 3 parameters are from the first hour of ICU stay. NUTRIC score was calculated as the modified version validated by Rahman et al. without interleukin-6 [4]. High nutritional risk was considered when the NUTRIC score had values ≥ 5 as previously defined in guidelines [2]. Frailty was defined as a Clinical Frailty Scale ≥ 5 [13].

Nutrition adequacy was calculated as the proportion of the caloric prescription received (either enterally or parenterally) during the first week of ICU admission and was considered adequate when patients received ≥ 80% of calories and protein prescribed. Goal of caloric intake was calculated according to the American guidelines [2]: 25 Kcal/kg body weight/day for patients with BMI < 30 or 14 Kcal/kg body weight/day for those patients with BMI ≥ 30. Goal of protein intake was 1.3 g/kg body weight /day. This analysis was restricted to patients whose data on the prescribed and infused calorie were available.

### SAPS-NUTRIC derivation

We derived SAPS-NUTRIC score from the previous one described by Rahman et al. [4]. Common variables were age, Sequential Organ Failure Assessment (SOFA), number of comorbidities and length of hospital stay before ICU admission. In order to convert APACHE II score into SAPS 3 as a substitute variable in SAPS-NUTRIC we first estimated the predicted range of probability of death for each stratum of APACHE II in NUTRIC score (i.e. less than 15; from 15 to 19; from 20 to 27; higher or equal to 28) without adjustments of admission reason. Then, SAPS 3 values with equivalent predicted probability of death were chosen (less than 53; from 53 to 57; from 58 to 74; higher or equal to 75). This procedure was chosen in order to maintain similar severity of illness prediction from the original NUTRIC derivation, but with a more accurate and updated score.

### Statistical analysis

Continuous variables were tested for normality using the Kolmogorov-Smirnov test. Continuous parametric variables were compared by unpaired t-test for analysis between groups and by paired t-test for paired analysis. Categorical variables were evaluated using the χ² test or Fisher’s exact test, when appropriate. Continuous nonparametric variables were evaluated using the Wilcoxon or Mann-Whitney tests.

Primary outcome was 28-day mortality (same primary outcome in the original study and validation studies) [3, 4]. To evaluate the predictive performance of NUTRIC and SAPS-NUTRIC score, we first compared discrimination between these models with the area under the Receiver Operating Characteristics (AUROC) curve by the Delong method [14]. Then, calibration was assessed by the calibration belt method as described by the GiViTI group [15]. This method applies a generalized polynomial logistic function between the outcome and the logit transformation of the estimated predicted probability, with the respective 95% confidence intervals (CI) boundaries. A statistically significant deviation from the bisector (the line of perfect calibration) occurs when the 95% CI boundaries of the calibration belt do not include the bisector [15]. Finally, Brier score as an overall performance measure was calculated using the standard formula [16].

Correlation between NUTRIC and SAPS-NUTRIC score was compared with intraclass correlation (2-way mixed effects model). Patients’ reclassification was analyzed with net reclassification index (NRI). Since mortality rate are not expected to be equal to survival rate (i.e. 50% to 50%) in our cohort, absolute NRI is expected to be more accurate than additive NRI [17]. Furthermore, the association between nutritional adequacy and 28-day mortality was assessed by stratifying the patients according to their nutritional risk group and compared with chi-squared test. Statistical analyzes were performed by using the software SPSS® Statistics 20 (IBM Corp., Armonk, New York, USA), STATA (StataCorp. 2017. Stata Statistical Software: Release 15. College Station, TX: StataCorp LLC) and R (http://www.r-project.org). Significant differences were stated at 5% level.

## Results

During the study period, we studied 542 patients; 282 (52%) were categorized as low and 260 (48%) as high nutritional risk according to the NUTRIC score (Table 1). Those patients categorized as high nutritional risk were older, more frequently admitted for non-surgical reasons, with higher prevalence of comorbidities and frailty compared to low nutritional risk patients. They also had higher severity-of-illness scores at ICU admission, required invasive organ support more frequently and had higher 28-day mortality compared to low risk patients (25.4% vs 11.4% respectively, p<0.001). Some differences could be observed between low and high nutritional risk groups regarding types of nutritional support (Table 2).

**Table 1:** Patient’s characteristics of included patients and stratified by NUTRIC score.

	All patients (n = 542)	Low risk* (n = 282)	High risk* (n = 260)	p Value^$^
Age (IQR), years	78 (65–86)	71 (59–84)	81 (73–88)	**< 0.001**
Male, n (%)	294 (54.2)	142 (50.4)	152 (58.5)	0.058
Body mass index (SD)	25.1 (5.2)	25.2 (5.2)	25.1 (5.1)	0.900
Unintentional Weight Loss, n (%)				0.492
No	441 (81.4)	226 (80.1)	215 (82.7)	
Yes, significant^&^	29 (5.4)	14 (5.0)	15 (5.8)	
Yes, severe^&&^	72 (13.3)	42 (14.9)	30 (11.5)	
Frailty, n (%)	310 (57.2)	135 (47.9)	175 (67.3)	**< 0.001**
Comorbidities, n (%)				
Diabetes	144 (26.6)	60 (21.3)	84 (32.3)	**0.004**
Neoplasia	188 (34.7)	109 (38.7)	79 (30.4)	**0.043**
Congestive heart failure	40 (7.4)	13 (4.6)	27 (10.4)	**0.010**
COPD	60 (11.1)	13 (4.6)	47 (18.1)	**< 0.001**
Chronic kidney failure	109 (20.1)	23 (8.1)	86 (33.1)	**< 0.001**
Dementia	137 (25.3)	54 (19.2)	83 (31.9)	**0.001**
LOS (days) before ICU admission ≥ 1, n(%)	193 (35.6)	77 (27.3)	116 (44.6)	**< 0.001**
Clinical admission, n (%)	398 (73.4)	195 (69.2)	203 (78.1)	**0.019**
Prognostic scores				
SAPS 3 (SD)	52 (18)	46 (21)	58 (11)	**< 0.001**
APACHE II (SD)	18 (7)	13 (5)	23 (5)	**< 0.001**
SOFA (SD)	5 (3)	3 (2)	7 (3)	**< 0.001**
Organ support during ICU stay, n(%)				
Vasoactive drugs	271 (50.0)	125 (44.3)	146 (56.2)	**0.006**
Mechanical ventilation	279 (51.5)	120 (42.6)	159 (61.2)	**< 0.001**
Dialysis	74 (13.7)	26 (9.2)	48 (18.5)	**< 0.001**
28-day mortality, n (%)	98 (18.1)	32 (11.4)	66 (25.4)	**< 0.001**

*Nutritional risk was defined as low (< 4 score) and high (≥ 5 score) by the NUTRIC Score [4].

$ p value for comparison between low and high risk groups. IQR, interquartile range; LOS: length of hospital stay; SD: standard deviation; COPD: Chronic Obstructive Pulmonary Disease; ICU: intensive care unit.

^&^Significant weight loss: ≤ 2% last week or ≤ 5% last month or ≤ 7.5% last 3 months or ≤10% last 6 months.

^&&^Severe weight loss: > 2% last week or > 5% last month or > 7.5% last 3 months or > 10% last 6 months.

**Table 2:** Nutritional support provided to included patients and stratified by NUTRIC score.

	All patients (n = 542)	Low risk* (n = 282)	High risk* (n = 260)	p Value^$^
Type of support, n(%)				**0.004**
Enteral nutrition	396 (73.1)	194 (68.8)	202 (77.7)	
Parenteral nutrition	81 (14.9)	56 (19.9)	25 (9.6)	
Enteral with parenteral nutrition	65 (12.0)	32 (11.4)	33 (12.7)	

*Nutritional risk was defined as low (< 4 score) and high (≥ 5 score) by the NUTRIC Score [4].

$ p value for comparison between low and high risk groups.

### Comparison between NUTRIC and SAPS-NUTRIC performance

Mean values of NUTRIC and SAPS-NUTRIC scores were 4.5 ± 1.9 and 4.1 ± 1.8 respectively. In our cohort, 326 (60.1%) were categorized as low and 216 (39.9%) as high nutritional risk according to the SAPS-NUTRIC score.

Our analysis showed that both NUTRIC and SAPS-NUTRIC demonstrated similar performance in discriminating 28-day mortality (p = 0.504), with adequate calibration evaluated by the calibration belt method. Brier scores were similar (Table 3 and S1 Appendix). Intraclass correlation between these two tools was 0.86 (CI 95% 0.83–0.88). Reclassification analysis demonstrated an additive NRI of -0.06 with an absolute NRI of 5.16% (S2 Appendix).

**Table 3:** Comparison of SAPS 3 performance in all patients and in subgroups of oncological and nononcological patients.

	Discrimination [AUROC (95% CI)]	Calibration*		Precision
		Over the bisector 95% CI	Under the bisector 95% CI	Brier score
NUTRIC	0.66 (0.61–0.73)	Never	Never	0.28
SAPS-NUTRIC	0.67 (0.63–0.71)	Never	Never	0.28

CI, confidence interval. AUROC, area under the receiver operating characteristic curve.

*Calibration described as the bisector deviation intervals by the calibration belt method.

### Nutritional adequacy and 28-day mortality

**Fig 1:**
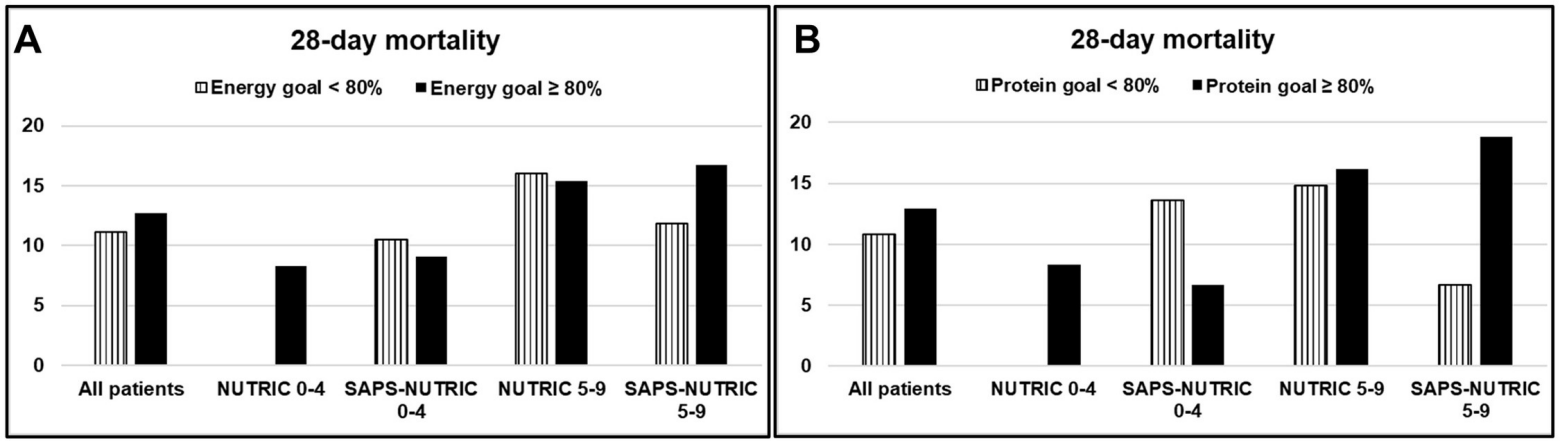
28-day mortality rate stratified by nutritional risk according to the adequacy of energy (A) and protein (B) received. No differences in 28-day mortality were observed in critically ill patients who achieved or not adequate energy (A) and protein supply (B), regardless their nutritional risk (p > 0.05 for each comparison).

For the 99 patients analyzed for nutritional adequacy, 35 patients (35.4%) were categorized as low nutritional risk and 64 patients (64.6%) as high nutritional risk, according to the NUTRIC score, and 52 patients (52.5%) were categorized as low nutritional risk and 47 patients (47.5%) as high nutritional risk, according to the SAPS-NUTRIC score. No differences in 28-day mortality could be observed between those groups whose energy or protein goals ≥ 80% of prescribed were achieved compared with those whose goals were not reached (Fig 1).

## Discussion

The main findings of the present study were: (1) the APACHE II replacement by SAPS 3 in NUTRIC score (SAPS-NUTRIC) had comparable performance in our single-center retrospective cohort to predict 28-day mortality, and (2) in a subgroup analysis, we did not observe an interaction between nutritional adequacy, NUTRIC or SAPS-NUTRIC categories and mortality.

Prevalence of high nutritional risk categorized by the NUTRIC score is variable in the literature (ranging from 32% to 57%) [5, 18–20], probably due to different case-mix in published cohorts. Therefore, discrimination to predict mortality described by ROC curves ranges between 0.64–0.79 [3–5, 18, 21]. It is reassuring that the ROC curves in our study are within these reported ranges and no difference was observed between NUTRIC and SAPS-NUTRIC score, suggesting similar discrimination. Of note, our results are similar to those reported in some of the largest multicenter validation studies (ROC curve of 0.65 in Rahman et al’ publication with 1199 patients [4] and 0.66 in Mendes et al’ publication with 1143 patients [5]) and a similar publication from Brazil (0.62 in Toledo et al’ publication) [21].

Calibration is another aspect of predictive performance that is relevant for new models, specially because it deteriorates over time [22–24]. Since Hosmer and Lemeshow goodness of fit test usually lacks statistical power to reject poor calibration [22], new methods to assess calibration have been described such as the calibration belt [15]. Both NUTRIC and SAPS-NUTRIC models had excellent calibration without any underestimation or overestimation in our cohort. This is relevant because calibration is considered the “most important property of a model” [17] and good calibration is required to properly evaluate reclassification. Our results with an absolute NRI of 5.16% suggest an improvement in reclassifying patients with SAPS-NUTRIC instead of NUTRIC model.

However, why should one bother to use the new SAPS-NUTRIC instead of the old NUTRIC score since their predictive performance showed similar results of discrimination, calibration and Brier scores? In fact, SAPS 3 can be calculated in the first hour of ICU admission with fewer variables while APACHE II can only be calculated after 24 hours, which might allow one to screen nutritional risk at admission and formulate a nutritional plan. Also, many units have adopted newer and updated prognostic scores instead of APACHE II (for examples, in Brazil the National Board of Intensive Care Medicine uses SAPS 3 in the National Registry of Intensive Care Units) [25]. Consequently, to calculate NUTRIC score this would demand calculation of two prognostic scores. Finally, performance between NUTRIC and SAPS-NUTRIC scores were similar with high intraclass correlation (suggesting good content agreement), but absolute NRI suggested some proper reclassification in a proportion of patients with SAPS-NUTRIC.

Our subgroup analysis did not demonstrate an association between 28-day mortality and energy-protein adequacy, even in those categorized as high nutritional risk by both tools. This finding is similar to those demonstrated by Arabi et al. who studied in a post-hoc analysis of the PermiT Trial the interaction between nutritional risk groups (stratified by NUTRIC score) and permissive underfeeding or standard feeding [26]. Similar outcomes considering risk groups were observed in their study, raising the question whether NUTRIC score, which has not been prospectively validated, is appropriate to identify who might benefit for early aggressive feeding and who might be harmed [27].

Globally, our results suggest that NUTRIC score may represent the severity of illness and predicted mortality, and are in accordance to a recent systematic review [28]. However, the utility of this tool, which does not incorporate anthropometric or nutritional-related variables, to screen nutritional risk in critically ill patients still need validation in prospective studies. Most of previous literature are from observational studies, which may suffer from confounding or indication bias. Even in the original publication of NUTRIC score the authors explained that the lack of nutritional variables was mainly due to difficulty to obtain these information in their database (>70% of missingness), a fact that might influence the inclusion of these variables in their model [3]. Thus, the utility of a prognostic score such as NUTRIC as a clinical decision tool should be further evaluated to avoid misapplication [29].

Our study has some limitations. First, our study is a single-center cohort, which may be influenced and biased by local practice and case-mix. However, our performance prediction analysis is in concordance with some previous studies and this is reassuring. Second, our nutritional adequacy analysis was performed in a smaller subgroup, what is prone to error type II and might explain the lack of association between energy-protein supply and outcomes. However, a post-hoc analysis of a large randomized controlled trial suggested the same results [26]. Third, our population had a median age of 78 years old. If our results are applicable to younger patients remains to be demonstrated. Nevertheless, Toledo et al. demonstrated similar findings in a younger population (mean age of 63) [21]. Finally, functional and health-related quality of life measures would be one of the most relevant outcomes in nutrition studies, but usually are secondary outcomes in large randomized controlled trials [30]. We do not have this information in our database, but we acknowledge the importance of this outcome for future studies in critical care nutrition, since like our study the other published validation cohorts of NUTRIC Score applied mortality as the primary outcome.

## Conclusions

In conclusion, in our cohort the performance of NUTRIC score with SAPS 3 (SAPS-NUTRIC) is similar to the original tool, with some improvement in reclassification. Therefore, it might be an option when APACHE II is not available. However, the utility of both tools to guide clinical decisions are yet to be demonstrated in further studies.

## Supporting information

S1 Appendix Calibration plot assessed by the calibration belt method. (DOCX)

S2 Appendix Risk reclassification tables. (DOCX)

S1 Dataset Dataset with anonymized information for primary analysis replication. (XLSX)

## List of abbreviations

NUTRIC score: NUTrition Risk in the Critically ill score
APACHE: Acute Physiology and Chronic Health Evaluation
SAPS 3: Simplified Acute Physiology Score 3
ROC: receiver operating characteristics curve
ICU: intensive care unit
SAPS-NUTRIC: NUTRIC after replacement of APACHE II by SAPS 3
BMI: body mass index
CI: confidence interval
NRI: net reclassification index
