# Supplementary material for: Performance of NUTRIC score to predict 28-day mortality in critically ill patients after replacing APACHE II with SAPS 3
Source: PLoS One. 2022 Jul 1;17(7):e0270455. doi: 10.1371/journal.pone.0270455 (PMC9249235; doi:10.1371/journal.pone.0270455)
Supplement: S1 Appendix — (DOCX) [file pone.0270455.s001.docx]

**S1 Appendix.** Calibration plot assessed by the calibration belt method.

**
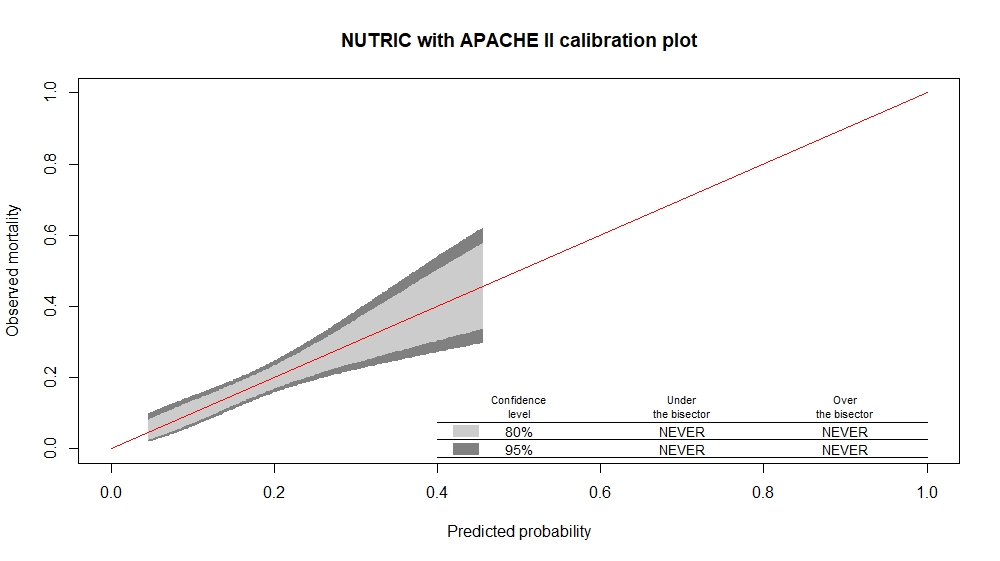
**

**
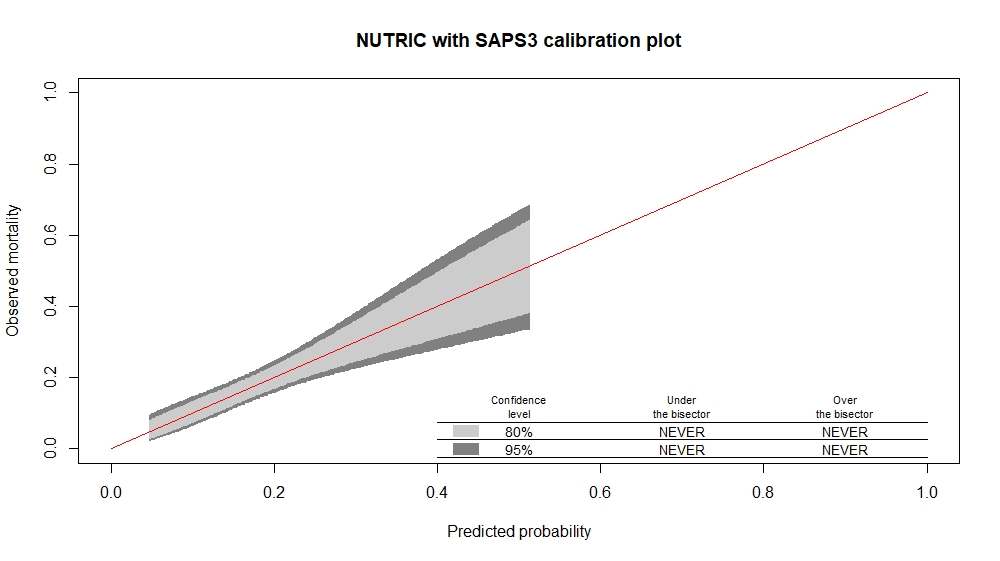
**

The predicted mortality intervals at which the calibration belt significantly deviates from the bisector and the 80% and 95% confidence levels are described in the lower right region part of the plots. The calibration belt contains the bisector (red line representing the identity between predicted probability and observed response rate) if the model is calibrated. If the belt does not include the bisector (is under or over the bisector), a significant prediction deviation is present in the model in the predicted range where this occurs.
