# Supplementary material for: Performance of NUTRIC score to predict 28-day mortality in critically ill patients after replacing APACHE II with SAPS 3
Source: PLoS One. 2022 Jul 1;17(7):e0270455. doi: 10.1371/journal.pone.0270455 (PMC9249235; doi:10.1371/journal.pone.0270455)
Supplement: S2 Appendix — (DOCX) [file pone.0270455.s002.docx]

**S2 Appendix.** Risk reclassification tables.

PATIENTS WITHOUT EVENT (n=444, 81.9%)

|  | | **SAPS-NUTRIC model** | |  |
| --- | --- | --- | --- | --- |
| **NUTRIC model** |  | **Low** | **High** | **Totals, NUTRIC model** |
|  | **Low** | 224 | 26 | 250 |
|  | **High** | 62 | 132 | 194 |
|  | **Totals, SAPS-NUTRIC model** | 286 | 158 | 444 |

PATIENTS WITH EVENT (n=98, 18.1%)

|  | | **SAPS-NUTRIC model** | |  |
| --- | --- | --- | --- | --- |
| **NUTRIC model** |  | **Low** | **High** | **Totals, NUTRIC model** |
|  | **Low** | 29 | 3 | 32 |
|  | **High** | 11 | 55 | 66 |
|  | **Totals, SAPS-NUTRIC model** | 40 | 58 | 98 |
